# Symptomatic Clitoral Neuroma within an Epidermal Inclusion Cyst at the Site of Prior Female Genital Cutting

**DOI:** 10.1155/2019/5347873

**Published:** 2019-07-29

**Authors:** Dani Zoorob, Kristrun Kristinsdottir, Thomas Klein, Sonyoung Seo-Patel

**Affiliations:** ^1^University of Toledo, Department of Obstetrics and Gynecology Academic Offices, 2142 N. Cove Blvd., Toledo, OH 43606, USA; ^2^ProMedica Health System, Women's Services, 2142 N. Cove Blvd., Toledo, OH 43606, USA

## Abstract

**Background:**

Clitoral neuromas occurring after female genital mutilation/cutting (FGM/C) can vary in presentation and may require surgical management.

**Case:**

A 39-year-old East African female with a history of FGM/C presented during pregnancy with a progressively enlarging mass and worsening periclitoral pain. Postpartum surgical excision restored cosmesis and resolved the discomfort with pathology confirming the presence of a neuroma within the epidermal inclusion cyst.

**Conclusion:**

We present the first published case of a symptomatic clitoral neuroma within an epidermal inclusion cyst. This unique pathology demonstrates that complications of female genital cutting can present in complex and varied ways. Considering the prevalence of FGM/C and increasing rates of emigration from countries in which FGM/C is performed, complex clitoral neuromas are an important long-term complication which providers in Africa or internationally must be aware of.

## 1. Introduction

Female genital mutilation/cutting (FGM/C) is sociocultural practice defined by the World Health Organization as the “partial or total removal of the external female genitalia or other injury to the female genital organs for nonmedical reasons” [1]. It is most commonly performed in parts of Africa, the Middle East, and Asia and frequently before the age of fifteen by a community leader. Analgesia is often not utilized, resulting in severe acute pain [1–3].

It is estimated that 100 to 140 million women around the world have undergone FGM/C, with approximately three million new cases each year [1, 4]. Recent data show decreasing acceptance of this practice even amongst countries where FGM/C is nearly universal [2].

In 2007, the WHO published a revised classification system to systematically describe the extent of FGM/C based on parts of the genitalia removed. Type I includes partial or total removal of the clitoris (clitoridectomy) while type II also includes removal of the labia minora with or without excision of the labia majora. Type III, also known as infibulation, is the most extensive form and includes narrowing of the vaginal orifice by appositioning a portion of the removed labia minora or majora. Type IV includes any other nonmedical procedures to female genitalia such as piercing, nicking, or cauterizing. Further subclassifications of FGM/C do exist and can be found in the literature [1].

Complications of FGM/C are many. Immediate complications include bleeding, infection, and pain [5, 6]. The vaginal tissue becomes more prone to tears, which increases the risk of acquiring sexually transmitted infections [7]. Women undergoing infibulation (Type III FGM/C) may be left with a small introitus, which can result in urinary tract infections and potentially prolonged, painful periods [5]. Additionally, the vagina may be scarred thus losing its elasticity, causing dyspareunia for both the patient and her partner, as well as obstetric complications during vaginal deliveries. Particularly with infibulation, women are at risk of prolonged labor and the need for cesarean section, postpartum hemorrhage, episiotomy, and prolonged maternal hospital stay. Their infants are at an increased risk of stillbirth and early neonatal death [8]. As the procedure often involves transection of the clitoris and the clitoral nerves, women are at risk of developing chronic localized pain and potentially neuroma formation [6]. Though lacking high-quality data, it is believed that the psychological consequences of this practice can be immense. A 2015 study of Senegalese women demonstrated a 30.4% prevalence of posttraumatic stress disorder attributed to this practice [8]. Thus, the practice of FGM/C has a multitude of short and long-term consequences.

## 2. Case

A 39-year-old G3P2002 female of Somali origin with two prior vaginal deliveries in Somalia presented with a periclitoral swelling. This had been slowly enlarging over the previous 10 years, but the change during this pregnancy was rapid an acute. She had a history of FGM/C Type IIIb (infibulation with removal and appositioning of the labia majora) at seven years of age without reported complications. Past medical and surgical history were otherwise unremarkable. The patient had undergone prior defibulation at delivery, which was not noted to be protracted nor required operative delivery. She denied any urinary symptoms and had expectantly managed her symptoms throughout the pregnancy. Surgical management was deferred until following delivery despite the persistent periclitoral burning pain that worsened as the lesion enlarged throughout the pregnancy. The patient also noted worsening dyspareunia symptoms as the pregnancy progressed even before fetal size caused appreciable pressure. The patient delivered via cesarean section due to breech presentation and she represented during her postpartum course for surgical removal of the lesion due to persistent symptoms which negatively impacted her quality of life.

**Figure 1:**
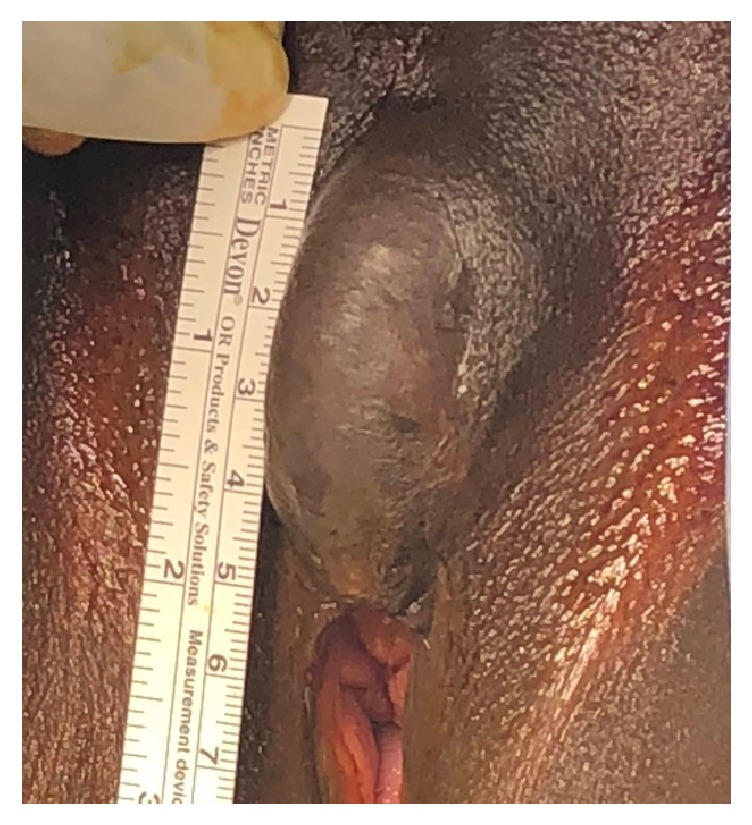
Preoperative view of the clitoral neuroma.

**Figure 2:**
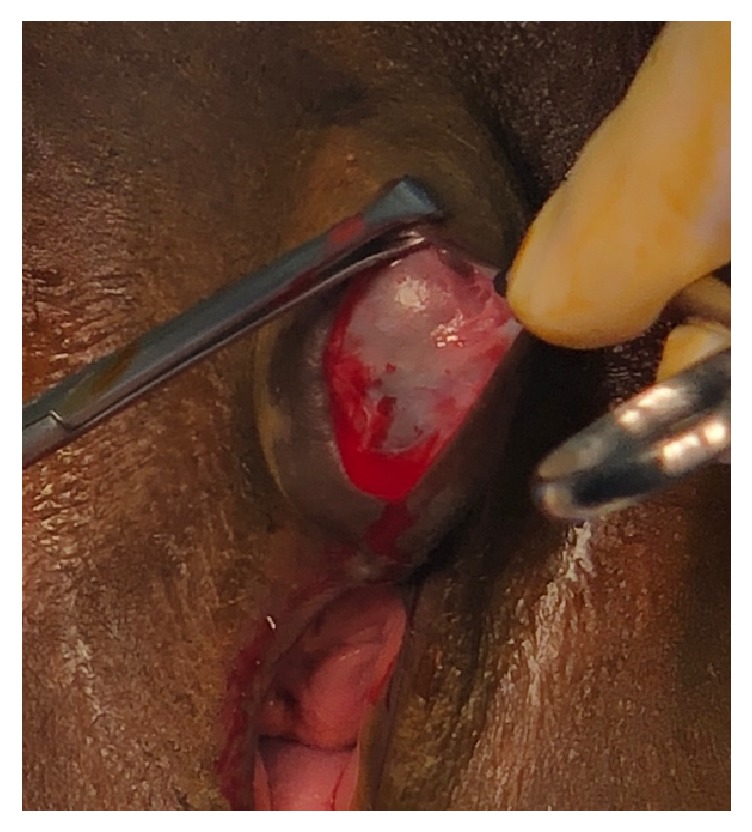
Cystic appearance of the neuroma.

Preoperatively, physical examination demonstrated the presence of a tender 4 cm x 3 cm x 2 cm periclitoral fluctuant lesion (Figure 1). Surgically, the lesion was treated by excision and reapproximation. This was performed by infiltrating the overlying skin with Marcaine, incising vertically at midline, and then dissecting around the cyst using both blunt and sharp dissection (Figure 2). After near complete shelling of the cyst, a stalk was noted to extend across the cyst. Due to the site of attachment and origin, it was suggestive of a clitoral remnant. The neuroma stalk was noted to be firm and thick on palpation. The stalk was transected at the base where the cyst wall originated and the cyst was freed. Hemostasis was ensured. A mattress suture was used to close the defect. Excess skin was trimmed and reapproximated using a subcuticular suture for restored cosmesis.

**Figure 3:**
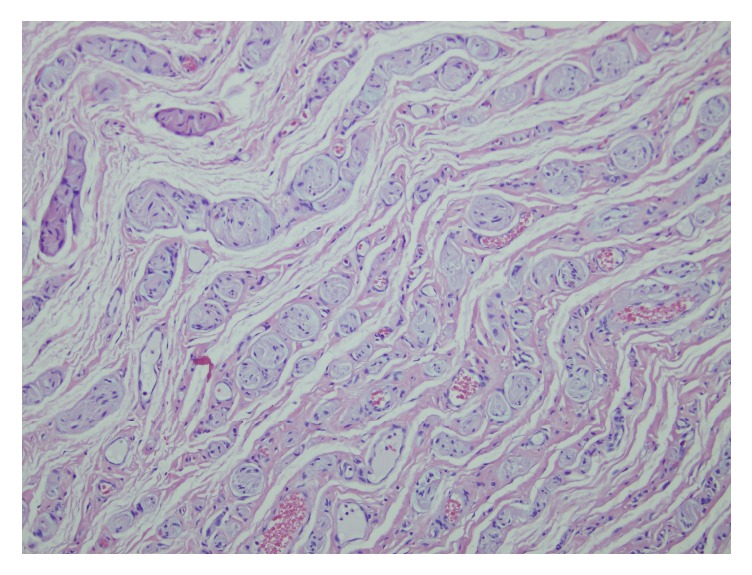
Microscopic view of the neuronal tissue identified within the cystic structure.

Following excision, the cyst was opened. Gross examination of the lesion demonstrated a dark grey stalk extending throughout two ends of the cyst with a glossy green-grey viscous material and no fluid. The widest diameter of the cyst measured 3.2 cm. Pathologic analysis of the cyst wall specifically demonstrated keratinizing squamous epithelium consistent with epidermal inclusion cyst. The stalk within the cyst wall was suggestive of a traumatic neuroma demonstrating proliferation of nerve fibers within a fibrotic tissue stroma (Figure 3). No malignancy was identified.

The patient's postoperative course was uncomplicated, and she reported resolution of both discomfort and dyspareunia.

## 3. Discussion

Relatively few clitoral neuromas have been reported as complications of FGM/C, but it is believed that this issue may be underrecognized and under-reported [5, 6, 9]. Clitoral neuromas after FGM/C are considered a type of amputation neuroma. We believe this is what contributed to our patient's condition. These entities arise after trauma to or transection of a nerve, and they are due to disorganized proximal nerve regeneration. Histopathology of the lesion typically shows irregularly arranged nerve fascicles within a fibrous stroma [5].

Treatment options include desensitization, steroid injections, and surgical excision, the latter of which is suggested to be the most efficacious therapy for resolution of traumatic clitoral neuromas. In such cases, recurrence of the traumatic neuroma is theoretically possible, and both providers and patients should be keenly aware of this possibility. However, to date, no cases have been published regarding such a complication [5]. In similar cases of traumatic penile neuromas, standard of care for symptomatic patients is surgical excision, which often results in full recovery [10, 11]. In the setting of FGM/C, the location and presentation of the stalk within the cyst are suggestive of a clitoral neuroma; however, the differential diagnosis does include a simple inclusion cyst, malignant growth of epidermal and mesenchymal tissue, and a secondary inclusion cyst within the original cyst.

Although few similar cases of neuromas have been published, none involved expectant patients [5]. Pregnancy-associated worsening of periclitoral pain—whose origin is the remnant of the dorsal nerve of the clitoris contained within an epidermal inclusion cyst—has never reported in the literature. Furthermore, this is the first case to demonstrate the presence of a clitoral neuroma contained within an epidermal inclusion cyst.

It is important to note that clitoral neuromas after FGM/C may not always be palpable, and the patient's presenting symptoms may only be allodynia or hyperalgesia. This pain is neuropathic and thus often described as a burning, tingling, or shooting sensation [6]. Although management for this patient would have remained the same whether or not the inclusion cyst contained the neuroma, the identification of neural involvement is clinically significant as it may require further treatment at a later point. Due to the variable clinical presentation and impact that clitoral neuromas can have on a patient's life, whether pain, discomfort, or perceived disfigurement, it is crucial that providers be aware of the potential sources of pain impacting patients who have undergone FGM/C and subsequent management options.

## Additional Points

*Precis. *Complications of female genital cutting require keen awareness of a variety of presentations and their subsequent management.* Teaching Points. *With increasingly diversified populations due to higher rates of global migration, women's healthcare providers should be aware of a variety of complications of female genital cutting. One important and likely under-reported complication is traumatic neuromas resulting in periclitoral neuropathic pain. Surgical excision has demonstrated great success and should be considered first-line management in such cases.
