# Corrigendum to “Symptomatic Clitoral Neuroma within an Epidermal Inclusion Cyst at the Site of Prior Female Genital Cutting”

**DOI:** 10.1155/2020/1240689

**Published:** 2020-09-02

**Authors:** Dani Zoorob, Thomas Klein, Kristrun Kristinsdottir, Sonyoung Seo-Patel

**Affiliations:** ^1^University of Toledo, Department of Obstetrics and Gynecology Academic Offices, 2142 N. Cove Blvd., Toledo, OH 43606, USA; ^2^ProMedica Health System, Women's Services, 2142 N. Cove Blvd., Toledo, OH 43606, USA

In the article titled “Symptomatic Clitoral Neuroma within an Epidermal Inclusion Cyst at the Site of Prior Female Genital Cutting” [1], the order of the second and third authors was reversed. The corrected author list is shown above.
